# Real-World Safety and Efficacy of Glycopyrronium Bromide in Japanese Patients with COPD: A 52-Week Post-Marketing Surveillance

**DOI:** 10.2174/18743064-v16-e2112240

**Published:** 2022-02-08

**Authors:** Chihiro Kato, Dong Wang, Noriko Nakamura, Takayoshi Sasajima, Hajime Yoshisue

**Affiliations:** 1 Clinical Development & Analytics, Novartis Pharma K.K., Tokyo, Japan; 2 Respiratory Medical Franchise, Novartis Pharma K.K., Tokyo, Japan

**Keywords:** Anticholinergic, Cardiovascular and cerebrovascular, Glycopyrronium, Long-term safety, Post-marketing surveillance, COPD

## Abstract

**Objective:**

To evaluate the long-term safety and efficacy of glycopyrronium (GLY) in patients with COPD in a real-world setting in Japan.

**Methods:**

This 52-week, multicentre, post-marketing surveillance conducted in Japan, between February 2013 and August 2019, included patients using GLY for the first time for the relief of airway obstructive disorder-related symptoms. Safety outcomes included incidence of adverse events (AEs), serious AEs (SAEs), adverse drug reactions (ADRs), serious ADRs (SADRs) and priority variables included cardiovascular/cerebrovascular (CCV) AEs and anticholinergic AEs during the 52-week period. Safety outcomes were also assessed in elderly patients. Efficacy outcomes included physician’s global assessment, COPD assessment test (CAT) and lung function test.

**Results and Discussion:**

Of the 1,331 patients registered for this surveillance, safety and efficacy outcomes were evaluated in 1,277 patients. In the safety analysis population, the incidence of AEs was 15.51%, SAEs 4.70%, ADRs 5.01% and SADRs 0.31%. The CCV AEs and anticholinergic AEs were reported by 0.70% and 2.58% patients, respectively. Physician’s global assessment showed that the overall response rate at the last assessment was 70%. The mean (95% CI) CAT scores decreased from the start of treatment to Week 52 with GLY, (−6.2 [−7.0 to −5.4]). Lung function in terms of trough FEV_1_ and FVC improved over time from the start of GLY to Week 52.

**Conclusion:**

GLY demonstrated an acceptable long-term safety profile with no new safety concerns in a real-life setting. It demonstrated improvement in lung function and symptom control in Japanese COPD patients.

## 1 INTRODUCTION

Chronic obstructive pulmonary disease (COPD) is characterized by persistent airflow limitation and respiratory symptoms that are due to airway and alveolar abnormalities usually caused by significant exposure to noxious particles or gases resulting in increased morbidity and a poor quality of life [1]. The Global prevalence of COPD was reported to be around 251 million cases in 2016 [2]. By 2030, COPD is projected to be the 3^rd^ leading cause of death worldwide [3]. The NIPPON COPD epidemiology (NICE) study reported that the prevalence of COPD in Japan is 8.6% in patients aged ≥40 years [4], although it is considered that the majority are not yet diagnosed.

The Global initiative for Chronic Obstructive Lung Disease (GOLD 2020) strategy recommends long-acting muscarinic antagonists (LAMAs) as the first-line therapy for patients with moderate-to-severe COPD (GOLD stages of B, C and D) [1]. In line with GOLD, the Japanese Respiratory Society guidelines also recommend treatment with LAMAs as the preferred treatment choice for patients across COPD severity (stage I-IV) [5].

Glycopyrronium bromide (GLY, a LAMA) inhibits acetylcholine-induced airway constriction by binding to muscarinic receptors in airway smooth muscle, thereby quickly improving respiratory function [6]. GLY is approved as a maintenance bronchodilator treatment to relieve symptoms in patients with COPD. It was approved in Japan in 2012, for “relieving the symptoms caused by airway obstructive disorder in COPD (chronic bronchitis and emphysema)” [7]. The approval in different countries worldwide and Japan was based on the Phase III GLOW trial programme comprising six clinical trials, which included patients with different ethnicities, and demonstrated the safety and efficacy of GLY in patients with COPD [8-13].

Data generated from clinical trials have demonstrated efficacy and safety of GLY in patients with stringent inclusion/exclusion criteria and, therefore, it may not fully represent the characteristics of a real-world setting. Limited evidence is available on safety and efficacy of GLY in real-world setting [14], especially in Japanese (Asian) patients with a different genetic makeup [15]. Data from real-world Japanese patients with COPD will help to validate the results generated from various RCTs and add to the existing evidence on safety and efficacy of GLY from randomized controlled trials (RCTs) in Japanese patients.

Upon approval, the Ministry of Health, Labour and Welfare (MHLW) identified the need to further assess the safety of GLY in the long term and elderly patients, anticholinergic adverse events (AEs) and cardiovascular/cerebrovascular (CCV) AEs. This post-marketing surveillance (PMS) was conducted to evaluate the long-term safety in terms of AEs, serious AEs (SAEs), adverse drug reactions (ADRs), and efficacy of GLY in Japanese patients with COPD.

## 2 METHODS

### 2.1 Study Design

This was a 52-week, multicenter, observational PMS, conducted from February 2013 to August 2019 in accordance with the Good Post-marketing Study Practice (GPSP) guidance. The surveillance included patients who were diagnosed as COPD and prescribed with GLY 50 μg o.d. (*via* the Breezhaler^®^ device) for the first time for relief of symptoms caused by airway obstruction disorder. The surveillance was conducted across 252 sites in Japan. The investigator/sub-investigator entered information on all registered patients from the start of GLY treatment to the completion/discontinuation of the observation period (12 weeks and 52 weeks) in the case report form (CRF) using an electronic data capture (EDC) system. The protocol of this surveillance was agreed upon in consultation with Pharmaceuticals and Medical Devices Agency (PMDA), and as such, informed consent from the patients was not mandated nor obtained.

### 2.2 Study Variables

#### 2.2.1 Patient Characteristics

Patient demographics and disease characteristics such as age, body mass index (BMI), COPD stage (JRS guideline [16]), dyspnea severity grade, and history of inhaled muscarinic antagonists or β_2_-agonists, complications, medical history and smoking status, were collected at the start of treatment in CRFs.

#### 2.2.2 Safety

Safety endpoints included incidence of AEs, ADRs, SAEs and serious ADRs (SADRs) during the 52-week observation period. AEs suspected by the investigator to be related to the study medication were classified as ADRs. Incidence of ADRs was also assessed by age subgroups.

The incidence of priority variables, which included CCV AEs and anticholinergic AEs, was also assessed. For CCV AEs and ADRs, the incidence and the incidence per 1,000 patient-years (PYs) were evaluated by the number of CCV disorder risk factors present. Safety was evaluated in the safety analysis population, which included patients whose CRFs were locked and excluded those meeting the criteria described in Table **S1**.

#### 2.2.3 Efficacy Endpoints

Efficacy endpoints included physician’s global assessment, COPD assessment test (CAT) and lung function test (spirometry). The physician’s global assessment (global impression of change) evaluated changes in the global clinical impression of patients on a 5-point scale (“excellent”, “good”, “moderate”, “poor” and “worsening”) from the start of GLY at Weeks 12 and 52. “Excellent”, “good”, and “moderate” were defined as response, whereas “poor”, “worsening”, or “not assessable” were considered as non-response. CAT scores and lung function (forced expiratory volume in one second [FEV_1_] and forced vital capacity [FVC]) were assessed at the start of GLY treatment and at Weeks 4, 12, 26, and 52, and at the last assessment time point. Efficacy was evaluated in the overall efficacy analysis population and by subgroups of COPD stages, body weight, BMI, dyspnea severity and elderly versus non-elderly population (Table **S2**).

### 2.3 Statistical Analysis

A sample size of 1,000 patients was considered adequate for this PMS, which would provide an adequate number of patients in different age groups, and therefore, the safety of GLY in elderly patients could be evaluated by segmented age groups. The period for the analysis of AEs (hereafter called the safety analysis period) is from the start date of GLY to ‘maximum observation period (52 weeks) + 30 days’, and for patients who discontinued/dropped out, the safety analysis period is from the start date of GLY to ‘last date of GLY administration + 30 days’. The number and proportion (incidence) of patients with AEs, ADR, SAEs and SADRs are summarised and calculated by system organ class (SOC) and preferred term (PT). For each of the CCV AEs, the number of applicable patients, patient years, incidence per 1,000 PYs, and the 95% confidence intervals (CIs) are calculated. The incidence of CCV AEs and CCV ADRs was also analyzed by a number of CCV disorder risk factors present. For spirometry (FEV_1_, FVC) and CAT, summary statistics are calculated for the respective assessment time points (at the start of GLY, Weeks 4, 12, 26, 52 of GLY treatment and at the last assessment), and presented as change over time in mean along with 95% CI values. Patients who skipped or had interrupted GLY treatment for 30 days or longer, were counted as discontinued, and their details were recorded in the discontinuation field.

## 3 RESULTS

### 3.1 Study Population

**Fig. (1):**
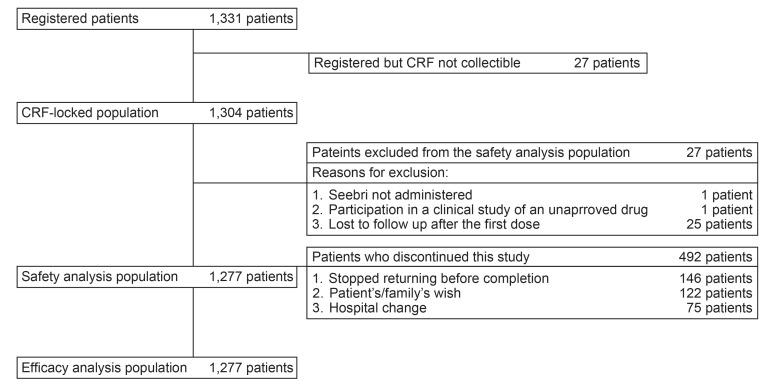
**Patient disposition** CRF, case report form.

In total, 1,331 patients registered for this surveillance and CRFs of 1,304 registered patients were locked. The safety and efficacy analysis population included a similar number of patients (n = 1,277), as none of patients in the safety analysis population met the exclusion conditions in the efficacy analysis population (Fig. **1**).

Baseline demographics and clinical characteristics of patients in the safety set are presented in Table **1.** Mean (± SD) age of patients was 73.1 (± 9.78) years, with 82.85% population aged ≥65 years. The majority of the patients had moderate COPD (stage II, 38.37%), followed by mild (stage I, 24.04%). Over 50% patients had prior treatment for COPD, with long-acting β_2_-agonists (LABA) (26.31%) accounting for the highest percentage followed by LAMA (19.34%) and LABA/inhaled corticosteroids (ICS) in 17.62% (17.62%). Mean (± SD) duration of GLY administration was 267.2 ± 134.59 days (median, 365.0 days).

### 3.2 Safety Outcomes

#### 3.2.1 Adverse events and Serious Adverse Events

Overall, 198 of 1,277 (15.51%) patients experienced AEs. The most frequently reported AEs by SOC were respiratory, thoracic and mediastinal disorders (7.05%), followed by cardiac disorders (2.66%), gastrointestinal disorders (2.51%), and infections and infestations (2.04%) (Table **2**). The most common AEs by PT (≥1% incidence) were COPD (4.46%), pneumonia (1.17%), and dry mouth (1.02%). In total, 60 (4.70%) patients reported SAEs. The most common SAEs by SOC were respiratory, thoracic and mediastinal disorders (2.04%), cardiac disorders (1.25%), and infections and infestations (1.02%) (Table **2**). By PTs, the most common SAEs (≥0.50% incidence) were COPD (1.41%), pneumonia (0.94%), and malignant lung neoplasm (0.55%).

#### 3.2.2 Incidence of ADRs and SADRs

Overall, 64 of 1,277 (5.01%) patients experienced ADRs. The most frequently reported ADRs by SOC were gastrointestinal disorders (1.72%), followed by respiratory, thoracic and mediastinal disorders (1.41%), and renal and urinary disorders (1.10%). By PT, the most common ADR (>1% incidence) reported was dry mouth (1.02%) (Table **3**). In total, 4 (0.31%) patients reported SADRs (Table **3**). The observed SADRs were respiratory tract infection, angina pectoris, atrial fibrillation, cardiac failure chronic, COPD, and interstitial lung disease in one patient each. All SADRs were resolved except for cardiac failure, where it proved to be fatal for one patient.

#### 3.2.3 ADR by Age Subgroups

Adverse drug reactions were not reported in any patients aged <45 years. However, the incidence of ADRs was the highest in patients aged ≥ 85 (7.03%). Incidence of ADRs was 4.76% in patients aged ≥45 to <55 years, 3.11% in patients aged ≥55 to <65 years, 5.30% in patients aged ≥65 to <75 years, and 5.04% in ≥75 to <85 years (Table **4**). Considering the ≥75 to <85-year age group (largest group) as the reference, there was no significant difference in the risk ratios of ADRs among the age subgroups. No specific trend in the types (by SOC) and incidence of adverse reactions by age groups was observed.

#### 3.2.4 Deaths

In total, 14 deaths were reported during the observation period. The fatal events due to AEs were three cases of COPD; two cases each for interstitial lung disease, malignant lung neoplasm and pneumonia; one case each of cancer pain, cardiac failure, congestive cardiac failure, chronic cardiac failure, squamous cell carcinoma of lung, esophageal carcinoma, aortic aneurysm rupture, myelodysplastic syndrome and concomitant disease progression. No event was considered to be related to GLY except for chronic cardiac failure. However, the patient had multiple underlying complications, which were adjudged by the physician as not associated with GLY.

#### 3.2.5 Anticholinergic Adverse Events and Adverse Reactions

The incidence of anticholinergic AEs in long-term treatment was 3.13%. The common AEs were dry mouth (1.02%), dysuria (0.94%) and constipation (0.55%), but no AE was serious in nature.

The incidence of anticholinergic adverse reactions in long-term treatment was 2.58%. The common ADRs were dry mouth (1.02%) and dysuria (0.78%); no ADR was serious in nature.

#### 3.2.6 CCV Adverse Events and Adverse Drug Reactions

The incidence of CCV AEs was 2.98%. The common AEs were cardiac failure in 0.55%, myocardial infarction in 0.39%, angina pectoris and ventricular extrasystoles in 0.31% patients each. In total, 19 serious CCV AEs were reported, all of which resolved except for 3 fatal cases (1 case each of cardiac failure, congestive cardiac failure, and chronic cardiac failure) and 2 unknown cases. The incidence per 1,000 PYs is described in Table **5**.

The incidence of CCV ADRs in long-term treatment was 0.70% (9/1,277 patients). Serious CCV ADRs occurred in 3 patients with one case each of angina pectoris, atrial fibrillation, and chronic cardiac failure. Of the three cases, one was resolved, one was resolving and the third case was left unresolved (chronic cardiac failure).

The incidence of CCV AEs increased with a higher number of CCV risk factors (Table **6**). In terms of CCV ADRs, the correlation between the incidence of reactions and CCV risk factors was not proportional. However, in patients with 3 or more risk factors, the incidence of adverse reactions was the highest (Table **6**).

### 3.3 Efficacy Endpoints

#### 3.3.1 Physician’s global assessment (global impression of change)

The physician’s global assessment results at the last assessment were “excellent” in 106 (8.30%), “good” in 490 (38.37%), “moderate” in 313 (24.51%), “poor” in 258 (20.20%) and “worsening” in 26 (2.04%) of patients. Considering “excellent”, “good” and “moderate” as response, the response rate in the overall efficacy analysis population was 71.18%. The response rates in global assessment at the last assessment in the respective COPD stage groups were 71.01% in patients with mild COPD, 71.63% in moderate COPD, 69.70% in the severe COPD group and 65.12% in the most severe group.

#### 3.3.2 COPD Assessment test (CAT) Score

**Fig. (2):**
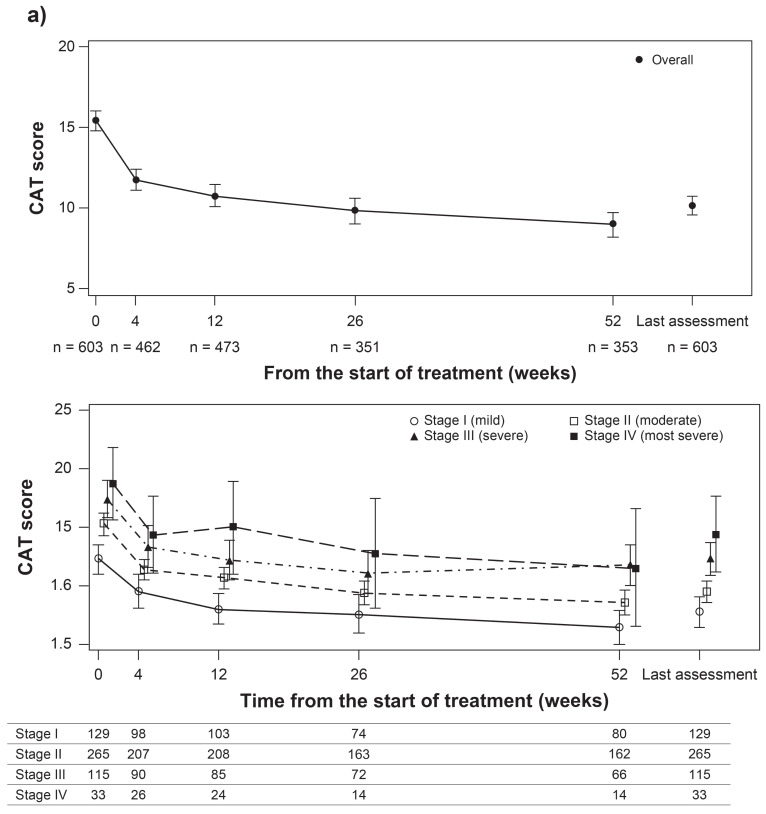
Change from baseline in CAT score in (a) overall population and b) by COPD stages (efficacy population). Data presented are mean (95% CI). CAT, Assessment test; CI, confidence interval; n, number of patients.

At the start of treatment with GLY, the mean (95% CI) CAT score was 15.4 (14.8 to 16.1) in the overall population. Treatment with GLY resulted in improvement in COPD symptoms as evidenced by a decrease in mean (95% CI) CAT score from the start of GLY treatment to Week 52 (9.0 [8.2 to 9.7]). The change in CAT score from baseline (start of GLY therapy) exceeded minimal clinically important difference (MCID) (≥2-point reduction) [17] at each evaluated time point until Week 52 (Fig. **2a**). By COPD severity, the mean (95% CI) CAT scores at the start of GLY and Week 52 were 12.3 (11.0 to 13.6) and 6.5 (5.0 to 7.9) in patients in stage I, 15.3 (14.3 to 16.2) and 8.6 (7.5 to 9.7) in stage II, 17.4 (15.8 to 19.0) and 11.8 (10.0 to 13.6) in stage III, and 18.7 (15.6 to 21.8) and 11.5 (6.5 to 16.5) in stage IV (Fig. **2b**).

#### 3.3.3 Lung Function for Overall Population and by COPD Severity

At the start of treatment with GLY, the mean (95% CI) FEV_1_ was 1.582 L (1.521 to 1.643), which increased over time from the start of therapy to Week 52. The mean (95% CI) change in FEV_1_ over time from the start of GLY was 0.177 L (0.122 to 0.232) at Week 52. By COPD stages, lung function improved over time from the start of treatment across COPD severity (Fig **S1**).

The mean (95% CI) FVC was 2.735 L (2.647 to 2.731) at the start of treatment and 2.922 (2.786 to 3.059) at Week 52. By COPD stages, the FVC improved over time from the start of GLY to Week 52 across all severity stages except stage IV. The changes over time in FEV_1_ and FVC for the overall population and by COPD stages are described in Fig (**S2**).

## 4 DISCUSSION

This PMS evaluated the long-term safety and efficacy of GLY during the 52 weeks from the start of treatment in clinical use in Japanese patients who were diagnosed with COPD and prescribed GLY for the first time. The results from this surveillance demonstrated that long-term treatment with GLY was not associated with an increase in the incidence of AEs and therefore considered to have an acceptable safety profile.

The efficacy and safety of GLY in patients with COPD are well established in numerous clinical trials [8-12], while few studies so far have evaluated the safety and efficacy of GLY in a real-world setting. In this surveillance, the incidence of AEs was low overall (15.51%). Common AEs were COPD, pneumonia and dry mouth. In a real-world study (GLARE) conducted in Taiwanese patients with COPD, 52.73% of patients in the study experienced AEs [14]. Patients were either treatment-naїve, received GLY as add-on, or switched to GLY in the GLARE study. In the 12-week, pragmatic CRYSTAL study, the incidence of AEs was 31.7% in COPD patients treated with GLY [18].

The overall safety profile observed in this surveillance was comparable to that observed in clinical studies evaluating GLY in COPD patients [10, 11, 13, 19]. The incidence of SAEs was 4.70% in this surveillance. The incidence of SAEs were 19.09% in the GLARE study, [14] and 2.3% in the CRYSTAL study [18]. The most common SAEs were COPD worsening followed by pneumonia. These were also the most common events in other clinical trials of GLY [9-11]. In this surveillance, the incidence of ADRs and SADRs was 5.01% and 0.31%, respectively.

Common ADRs in this study were dry mouth and dysuria. A 52-week parallel-group study (GLOW 4), conducted in Japanese COPD patients, assessed long-term safety and tolerability of GLY and showed similar adverse reactions [11]. Overall, no new safety concerns were observed in this study and the safety profile of GLY was consistent with that observed in the clinical trials, irrespective of the patient population, *i.e*. across ethnicities [11-13]. Although careful consideration should be given when comparing results from this surveillance with those from RCTs conducted under standardized conditions with fewer patients and strict enrolment criteria, the observational nature of this study enabled us to collect long-term safety and efficacy data from a larger pool of patients in a more realistic clinical setting.

The current surveillance also evaluated the incidence of CCV AEs and anticholinergic AEs, which were designated as priority variables for the purpose of this study. The commonly observed CCV AEs in this surveillance were cardiac failure, myocardial infarction, angina pectoris and ventricular extrasystoles.

A European multinational, multi-database (five European electronic health care databases) cohort study evaluated CCV AEs in COPD patients treated with GLY compared to LAMA or LABA. The incidence of CCV AEs was lower in the present study than in the European multinational cohort study. The incidence per 1,000 PYs was 8.5 and 3.86 for ischemic heart disease, 20.6 and 15.52 for cardiac arrhythmia, 9.9 and 2.89 for cerebrovascular disorder, 42.8 and 13.48 for death, in the cohort study and the present surveillance, respectively (Novartis data on file, NVA237A2402). The incidence of CCV AEs increased, along with the increase in risk factors, but a similar trend was not observed with CCV ADRs with the highest incidence of CCV ADRs observed in patients with 3 or more risk factors. The anticholinergic AEs, such as dry mouth and constipation observed in this surveillance were in line with those commonly associated with LAMAs, which were also reported in previous clinical trials of GLY [18, 19]. COPD is a disease of elderly patients. In Japan, the average age of patients diagnosed with COPD is around 70 years, as reported by several cohort studies [20-22]. The patient population included in this current surveillance was in line with the above findings, with 82.85% of the overall surveillance population aged ≥65 years. The age strata of ≥75 to <85 years comprised the largest patient population in this surveillance. There was no difference in the safety profile of GLY in the elderly patients compared with the overall surveillance population. The incidence of adverse reactions in the late elderly aged ≥75 years was 5.45% (34/624 patients), which was comparable to the 4.59% (30/653 patients) rate observed in the patients aged <75 years. The common ADRs observed in these patients include dry mouth, dysuria and cough. In general, the adverse reactions were balanced across the different age subgroups for all populations, signifying no prominent age-related difference in safety profile.

The efficacy endpoints evaluated in the study further validate the benefits observed with clinical studies and other real-world studies of GLY. In terms of physician’s global assessment, 71.18% of patients were found to be treatment responders, implying appropriateness of the GLY therapy in these COPD patients. A consistent improvement in lung function was observed over time with GLY, as evident from improvement in FEV_1_ and FVC. The mean change in CAT score from the start of GLY therapy at Week 52 was −6.2; the mean changes at all other evaluated time points from the start of treatment were greater than MCID, showing improvement in symptoms of COPD with GLY.

These results are consistent with the results observed in the previous clinical trial with GLY [23]. Consistent with the overall population, there was a decrease in CAT score over the study period with GLY treatment for different COPD stages (I–IV), including patients in stage I who are rarely enrolled in conventional clinical trials on COPD.

The surveillance has a few potential limitations. In line with other non-interventional observational studies, no control group was included; therefore, the results need to be interpreted carefully. Because of the nature of the surveillance in clinical use, consideration should be given to the fact that the spirometry conditions were different from patient to patient and that spirometry was not performed at trough (*e.g*., before the use of bronchodilators). The results of this study should be cautiously generalized to other ethnic populations as it was conducted in Japan and included Japanese patients with COPD.

## CONCLUSION

In this 52-week long-term, observational study, GLY demonstrated an acceptable safety profile in patients with COPD from Japan. No new safety concerns were identified with the use of GLY in the clinical setting, nor was the incidence of any ADR increased during the long-term treatment. The long-term safety profile of GLY in elderly patients and terms of CCV AEs and anticholinergic AEs did not show an increase in the incidence of AEs. Long-term treatment with GLY in COPD patients showed improvements in lung function and symptoms, regardless of the COPD stage, including stage I (patients with mild COPD).

## Figures and Tables

**Table 1:** Patient demographics and baseline characteristics (safety data set).

**Characteristics**	**Total number of patients (N = 1277)**
**Age, years** Mean ± SD Median (min – max)	73.1 ± 9.78 74.0 (29.0 – 94.0)
**Weight, kg** Mean ± SD Median (min – max)	58.3 ± 11.65 58.0 (26.0 − 105.0)
**BMI, kg/m^2^** Mean ± SD Median (min – max)	22.3 ± 3.85 22.3 (12.5 − 40.6)
**Smoking status, n (%)** Never smoked Ex-smoker Current smoker	183 (14.33) 721 (56.46) 265 (20.75)
**COPD duration, years,** Mean ± SD Median (min – max) <1 year, n (%) ≥1 to <3 years, n (%) ≥3 years, n (%)	3.9 ± 4.47 2.0 (0.00 − 15.00) 240 (18.79) 101 (7.91) 262 (20.52)
COPD stages, n (%)* Stage I (mild) Stage II (moderate) Stage III (severe) Stage IV (very severe)	307 (24.04) 490 (38.37) 231 (18.09) 86 (6.73)
**Dyspnea severity, (grade), n (%)^†^** 0 1 2 3 4	160 (12.53) 539 (42.21) 327 (25.61) 203 (15.90) 48 (3.76)
**Complications, n (%)** Bronchial asthma CCV disorder Hepatic disorder	929 (72.75) 288 (22.55) 239 (18.72) 39 (3.05)
**Other complications, n (%)** Yes No	425 (33.28) 852 (66.72)
**Prior medication for COPD, n (%)** SAMA LAMA SABA LABA ICS OCS.CSI LABA/ICS LABA/LAMA Others	7 (0.55) 247 (19.34) 24 (1.88) 336 (26.31) 37 (2.90) 18 (1.41) 225 (17.62) 0 (0.00) 325 (25.45)

*JRS Guidelines for the Management of COPD (version 3) (Please refer to Supplementary Table (**3**) for disease staging); †British Medical Research Council (MRC)

CCV, cerebrovascular/cardiovascular; COPD, chronic obstructive pulmonary disorders; CSI, corticosteroid injection; ICS, inhaled corticosteroid; LABA long-acting β_2_-agonists; LAMA, long-acting muscarinic antagonist; OCS, oral corticosteroid; SD, standard deviation; SABA, short acting β_2_-agonists; SAMA, short-acting muscarinic antagonist.

**Table 2:** Adverse events and serious adverse events by system organ class and preferred term (safety analysis population).

**SOC/PT**	**Total number of patients (N = 1277)**	
	**AEs ** **n (%)**	**SAEs ** **n (%)**
**Patients experiencing events**	**198 (15.51)**	**60 (4.70)**
Infections and infestations	26 (2.04)	13 (1.02)
Pneumonia	15 (1.17)	12 (0.94)
Neoplasms benign, malignant and unspecified	12 (0.94)	12 (0.94)
Immune system disorders	1 (0.08)	-
Metabolism and nutrition disorders	2 (0.16)	-
Psychiatric disorders	2 (0.16)	-
Nervous system disorders	8 (0.63)	2 (0.16)
Cardiac disorders	34 (2.66)	16 (1.25)
Vascular disorders	7 (0.55)	1 (0.08)
Respiratory, thoracic and mediastinal disorders	90 (7.05)	26 (2.04)
COPD	57 (4.46)	18 (1.41)
Gastrointestinal disorders	32 (2.51)	1 (0.08)
Dry mouth	13 (1.02)	-
Hepatobiliary disorders	2 (0.16)	-
Skin and subcutaneous tissue disorders	3 (0.23)	-
Musculoskeletal and connective tissue disorders	3 (0.23)	-
Renal and urinary disorders	19 (1.49)	1 (0.08)
Reproductive system and breast disorders	2 (0.16)	-
General disorders and administration site conditions	10 (0.78)	2 (0.16)
Concomitant disease progression	1 (0.08)	1 (0.08)
Investigations	9 (0.70)	1 (0.08)
Injury, poisoning and procedural complication	3 (0.23)	1 (0.08)

Data represented as n (%).

COPD, chronic obstructive pulmonary disorder; PT, preferred term; SOC, system organ class.

**Table 3:** Adverse reactions and serious adverse reactions by system organ class and preferred term (safety analysis population).

**SOC/PT**	**Total number of patients (N = 1277)**	
	**ADRs** **n (%)**	**SADRs** **n (%)**
**Patients experiencing drug reactions**	**64 (5.01)**	**4 (0.31)**
Infections and infestations	2 (0.16)	1 (0.08)
Nasopharyngitis	1 (0.08)	-
Respiratory tract infection	1 (0.08)	1 (0.08)
Nervous system disorders	1 (0.08)	-
Visual field defect	1 (0.08)	-
Cardiac disorders	9 (0.70)	3 (0.23)
Palpitations	2 (0.16)	-
Ventricular extrasystoles	2 (0.16)	-
Angina pectoris	1 (0.08)	1 (0.08)
Atrial fibrillation	1 (0.08)	1 (0.08)
Cardiac failure chronic	1 (0.08)	1 (0.08)
Myocardial infarction	1 (0.08)	-
Tachycardia	1 (0.08)	-
Respiratory, thoracic and mediastinal disorders	18 (1.41)	2 (0.16)
Cough	7 (0.55)	-
COPD	3 (0.23)	1 (0.08)
Dysphonia	3 (0.23)	-
Interstitial lung disease	1 (0.08)	1 (0.08)
Productive cough	1 (0.08)	-
Upper respiratory tract inflammation	1 (0.08)	-
Gastrointestinal disorders	22 (1.72)	-
Dry mouth	13 (1.02)	-
Constipation	6 (0.47)	-
Abdominal distension	2 (0.16)	-
Gastroesophageal reflux disease	1 (0.08)	-
Stomatitis	1 (0.08)	-
Skin and subcutaneous tissue disorders	1 (0.08)	-
Eczema	1 (0.08)	-
Renal and urinary disorders	14 (1.10)	-
Dysuria	10 (0.78)	-
Urinary retention	3 (0.23)	-
Nocturia	2 (0.16)	-
Reproductive system and breast disorders	2 (0.16)	-
Benign prostatic hyperplasia	2 (0.16)	-
General disorders and administration site conditions	3 (0.23)	-
Thirst	2 (0.16)	-
Chest discomfort	1 (0.08)	-

Data represented as n (%)

ADRs, adverse drug reactions; PT, preferred term; SADRs, serious adverse drug reactions, SOC, system organ class.

**Table 4:** Data on the occurrence of adverse drug reactions by system organ class and age category (safety analysis population).

**Characteristics**	**Age subgroups, years**					
	**<45**	**≥45 to <55**	**≥55 to <65**	**≥65 to <75**	**≥75 to < 85**	**≥85**
Number of patients	16	42	161	434	496	128
Incidenceof ADRs	-	4.76	3.11	5.30	5.04	7.03
Risk ratio	-	0.94	0.62	1.05	Reference	1.40
95% CI for risk ratio	-	0.23 to3.85	0.24 to1.58	0.61 to1.83	-	0.67 to2.91
**Type of adverse drug reaction by SOC**	**No. of applicable patients (%)**					
Infections and infestations	-	-	-	-	2 (0.40)	-
Nervous system disorders	-	-	-	-	1 (0.20)	-
Cardiac disorders	-	-	-	3 (0.69)	3 (0.60)	3 (2.34)
Vascular disorders	-	-	1 (0.62)	-	-	-
Respiratory, thoracic and mediastinal disorders	-	1 (2.38)	1 (0.62)	6 (1.38)	7 (1.41)	3 (2.34)
Gastrointestinal disorders	-	1 (2.38)	2 (1.24)	8 (1.84)	9 (1.81)	2 (1.56)
Skin and subcutaneous tissue	-	-	-	-	1 (0.20)	-
Renal and urinary disorders	-	-	1 (0.62)	6 (1.38)	4 (0.81)	3 (2.34)
Reproductive system and breast disorders	-	-	-	2 (0.46)	-	-
General disorders and administrationsite conditions	-	-	-	3 (0.69)	-	-

Incidence of adverse reactions by age category (safety analysis population).

System organ class SOC: MedDRA/J Version 22.0.

**Table 5:** Data on occurrence of CCV adverse events (patient-year method) (safety analysis population).

	**Total no of patients (N = 1277)**		
	**No. of patients experiencing CCV AEs**	**PY**	**IR (95% CI)**
CCV disorder	38	1,023	37.13 (26.28 to 50.97)
Ischaemic heart disease	4	1,037	3.86 (1.05 to 9.88)
Cardiac arrhythmia	16	1,031	15.52 (8.87 to 25.21)
Cerebrovascular disorder	3	1,039	2.89 (0.60 to 8.44)
Death	14	1,038	13.48 (7.37 to 22.62)

*IR per 1,000 PY

Multiple episodes of an event in the same patient are counted only once for the first occurrence. AE, adverse event; CI, confidence interval; CCV, cardiovascular/cerebrovascular; IR, incident rate; PY, patient-year.

**Table 6:** Incidence of CCV adverse events and adverse drug reactions by a number of CCV disorder risk factors (safety analysis population).

	**AEs**		**ADRs**	
	No. of patients with CCV AEs/no. of cases (%)	No. of patients with CCV AEs/PY (IR)	No. of patients with CCV ADRs/no. of cases (%)	No. of patients with CCV ADRs/PY (IR)
CCV history: yes	19/273 (6.96)	19/206 (92.09)	5/273 (1.83)	5/213 (23.51)
CCV risk factors: 0	1/59 (1.69)	1/44 (22.58)	0/59 (0.00)	0/45 (0.00)
CCV risk factors: 1	7/313 (2.24)	7/254 (27.55)	1/313 (0.32)	1/256 (3.91)
CCV risk factors: 2	10/258 (3.88)	10/214 (46.76)	0/258 (0.00)	0/218 (0.00)
CCV risk factors: ≥ 3	12/271 (4.43)	12/216 (55.43)	4/271 (1.48)	4/221 (18.10)

Incidence of CCV AEs and CCV adverse reactions by number of CCV disorder risk factors present (safety analysis population)

CCV risk factors include: (1) history of CCV disorder, (2) hypertension, (3) hyperlipidaemia, (4) diabetes mellitus, (5) BMI: >30 kg/m^2^, (6) age ≥65 years, (7) current smoker. ADRs, adverse drug reactions; AE, adverse event; BMI, body mass index; CCV, cardiovascular/cerebrovascular; IR, incident rate (per 1,000*PY); PY, patient-year.

## Data Availability

All data relevant to the study are included in the article or uploaded as supplementary information.
