# Supplementary material for: Real-World Safety and Efficacy of Glycopyrronium Bromide in Japanese Patients with COPD: A 52-Week Post-Marketing Surveillance
Source: Open Respir Med J. 2022 Feb 8;16:e187430642112240. doi: 10.2174/18743064-v16-e2112240 (PMC10156031; doi:10.2174/18743064-v16-e2112240)
Supplement: Supplementary file 1 [file TORMJ-16-e187430642112240_SD1.pdf]

1874-3064/22 *Send Orders for Reprints to reprints@benthamscience.net*

The Open Respiratory Medicine Journal

Content list available at: https://openrespiratorymedicinejournal.com

Supplementary Material

Real-World Safety and Efficacy of Glycopyrronium Bromide in Japanese Patients with COPD: A 52-Week Post-Marketing Surveillance

Chihiro Kato[1], Dong Wang[1], Noriko Nakamura[1], Takayoshi Sasajima[1] and Hajime Yoshisue[2,*]

[1]*Clinical Development & Analytics, Novartis Pharma K.K., Tokyo, Japan*
[2]*Respiratory Medical Franchise, Novartis Pharma K.K., Tokyo, Japan*

Article History	Received: July 25, 2021	Revised: November 10, 2021	Accepted: November 17, 2021

Lung Function

Forced Expiratory Volume in One Second (FEV_1)

The mean (95% CI) FEV_1 at start of GLY treatment was 1.582 L (1.521 to 1.643) in the overall study population. Treatment with GLY improved lung function with mean (95% CI) FEV_1 of 1.704 L (1.618 to 1.789) at Week 4, 1.749 L (1.662 to 1.835) at Week 12, 1.786 L (1.674 to 1.899) at Week 26, and 1.804 L (1.699 to 1.908) at Week 52 (Fig. **S1a**). Data presented are mean (95% CI); CI, confidence interval; L, liter; n, number of patients

By disease stage, the FEV_1 results [mean (95% CI)] at the start of GLY and Week 52 were 2.027 L (1.903 to 2.151 L) and 2.147 L (1.904 to 2.390 L) in the stage I group, 1.656 L (1.589 to 1.722 L) and 1.892 L (1.772 to 2.012 L) in the stage II group, 1.049 L (0.974 to 1.125 L) and 1.268 L (1.082 to 1.454 L) in the stage III group, and 0.829 L (0.657 to 1.001 L) and 1.058 L (0.573 to 1.543 L) in the stage IV group (Fig. **S1b**).

Table S1. Safety analysis exclusion conditions.

Exclusion conditions	Definitions
Registration unconfirmed	Patients whose registration form was not confirmed
Registered but not within the registration period	Patients deviated from the registration criteria
Treatment not started within the contract term	Patients who did not start treatment with GLY within the contract term, or within the protocol-specified registration period
GLY not administered	Patients confirmed after CRF collection not to have taken GLY
GLY-experienced	Patients in whom prior medication information (started before the start of GLY) includes GLY itself
Participation in a clinical study of an unapproved drug	Patients with information on study drugs that cannot be coded
Off-label use	Patients whose reason for GLY use is not “COPD”
Not within the contract term	Patients whose contract term expired during the observation period of CRF volume 1 (up to 12 weeks from the start of GLY, hereafter called volume 1)
Previous volume not locked	Patients whose CRF volume 2 (Week 13 to Week 52, hereafter called volume 2) is locked, but CRF volume 1 is not.
Lost to follow up after the first dose	Patients lost to follow up after the start day of GLY

Table S2. Efficacy analysis exclusion conditions.

Exclusion conditions	Definitions
Primary efficacy evaluation missing/not recorded	Patients whose “global assessment” in the last collected volume was left blank, making them not evaluable for global impression of change

DOI: 10.2174/18743064-v16-e2112240, 2022, *16*, e187430642112240-SP

Table S3. COPD Disease staging.

Stage	Characteristics
Stage I (Mild airflow obstruction)	FEV_1 / FVC < 70%<br>%FEV_1 ≥ 80%
Stage II (Moderate airflow obstruction)	FEV_1 / FVC < 70%<br>50% ≤ %FEV_1 < 80%
Stage III (Severe airflow obstruction)	FEV_1 / FVC < 70%<br>30% ≤ %FEV_1 < 50%
Stage IV (Very severe airflow obstruction)	FEV_1 / FVC < 70%<br>%FEV1 < 30%, or %FEV1 < 50% AND concurrent chronic respiratory failure
**FEV_1, forced expiratory volume in one second; FVC, forced vital capacity**	

a)

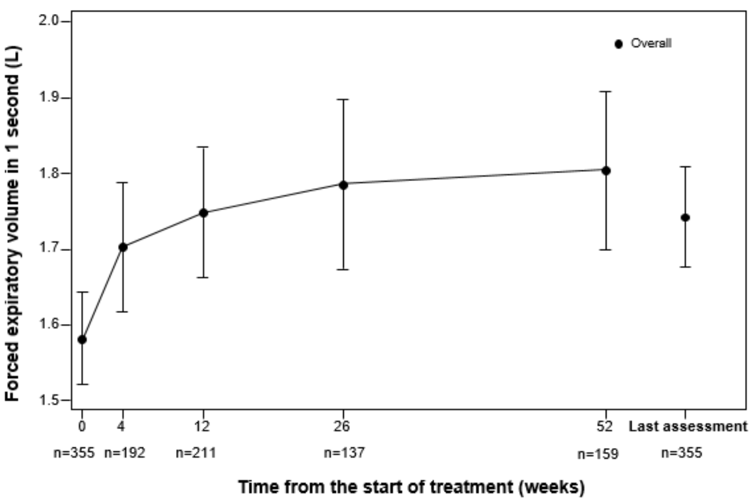

b)

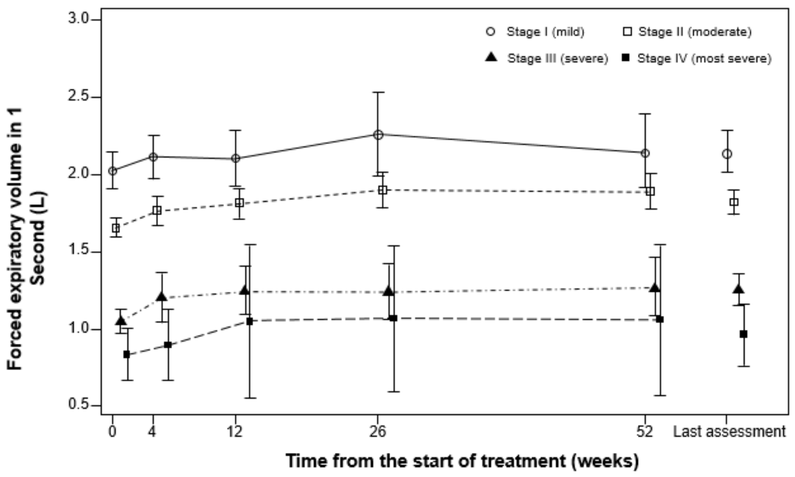

Stage I, n	85	52	53	28	37	85
Stage II, n	176	92	110	68	84	176
Stage III, n	76	38	41	33	32	76
Stage IV, n	14	9	6	6	5	14

Fig. (S1). Changes over time in FEV_1 score **a**) Overall population and **b**) by COPD stages (efficacy analysis population)

Forced Volume Capacity (FVC)

The mean (95% CI) FEV_1 at start of GLY treatment was 2.735 L (2.647 − 2.823) in the overall study population. Treatment with GLY improved lung function with mean (95% CI) FVC of 2.835 L (2.807 − 3.050) at Week 4, 2.850 L (2.760 − 2.990) at Week 12, 2.890 L (2.781 to 3.083) at Week 26, and 2.890 L (2.786 to 3.059) at Week 52 (Fig. **S2a**).

Data presented are mean (95% CI); n, number of patients, CI, confidence interval; L, litre.

a)

b)

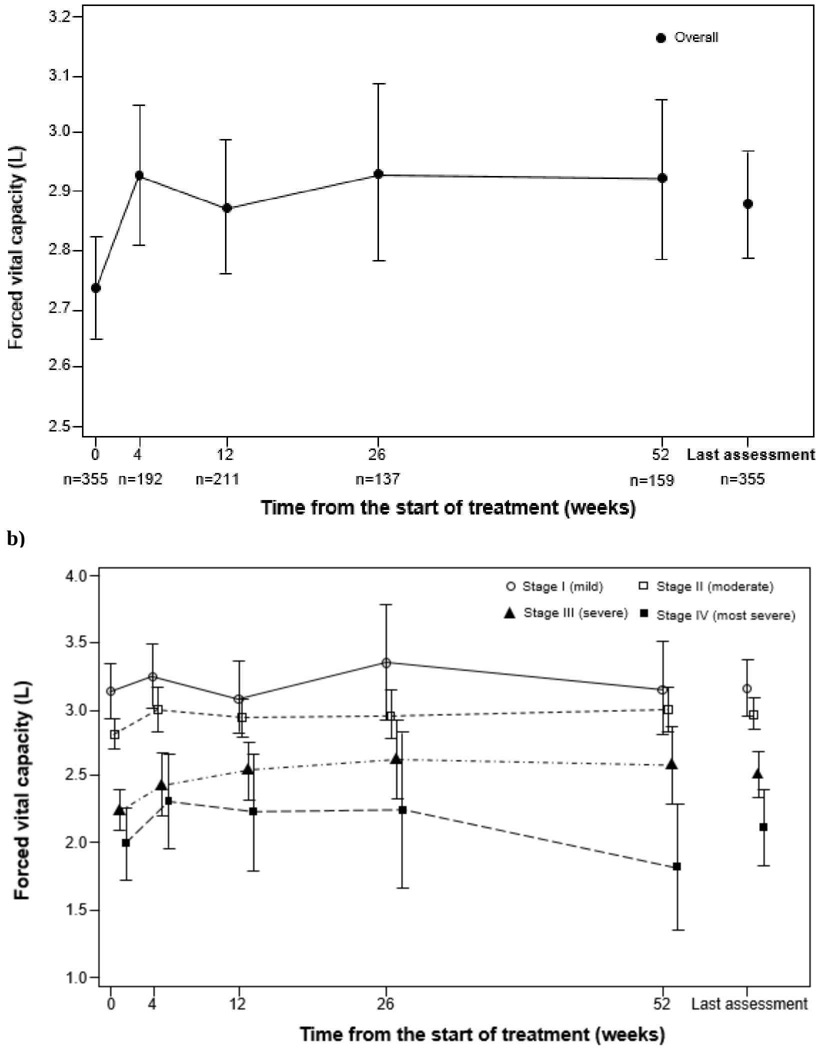

Stage I, n	85	52	53	28	37	85
Stage II, n	176	92	110	68	84	176
Stage III, n	76	38	41	33	32	76
Stage IV, n	14	9	6	6	5	14

Fig. (S2). Changes over time in FVC score **a**) Overall and **b**) by COPD stages (efficacy analysis population).

By disease stage, the FVC results [mean (95% CI)] at the start of GLY and Week 52 were 3.137 L (2.932 to 3.342 L) and 3.161 L (2.808 to 3.513 L) in the stage I group, 2.822 L (2.710 to 2.934 L) and 3.011 L (2.849 to 3.173 L) in the stage II group, 2.248 L (2.101 to 2.395 L) and 2.587 L (2.294 to 2.879 L) in the stage III group, and 1.996 L (1.722 to 2.271 L) and 1.820 L (1.346 to 2.294 L) in the stage IV group (Fig. **S2b**).

© 2022 Kato *et al*.

This is an open access article distributed under the terms of the Creative Commons Attribution 4.0 International Public License (CC-BY 4.0), a copy of which is available at: https://creativecommons.org/licenses/by/4.0/legalcode. This license permits unrestricted use, distribution, and reproduction in any medium, provided the original author and source are credited.
